# Supplementary material for: Characterization of the Glucan-Branching Enzyme GlgB Gene from Swine Intestinal Bacteria
Source: Molecules. 2023 Feb 16;28(4):1881. doi: 10.3390/molecules28041881 (PMC9960391; doi:10.3390/molecules28041881)
Supplement: Supplementary file 1 [file molecules-28-01881-s001.zip › Online supplementary material revised.pdf]

Online supplementary material to

Characterization of the glucan-branching enzyme *glgB* gene from swine intestinal bacteria and its potential role in forming low-digestible oligosaccharides

Yuqi Shao, Weilan Wang, Ying Hu, Michael G. Gänzle

Supplementary Figure S1. HPAEC-PAD profiles of oligosaccharides after hydrolysis of starch from pea

Supplementary Table S1. Concentration of iodine-binding amylose in raw starches before and after the treatment with GlgB.

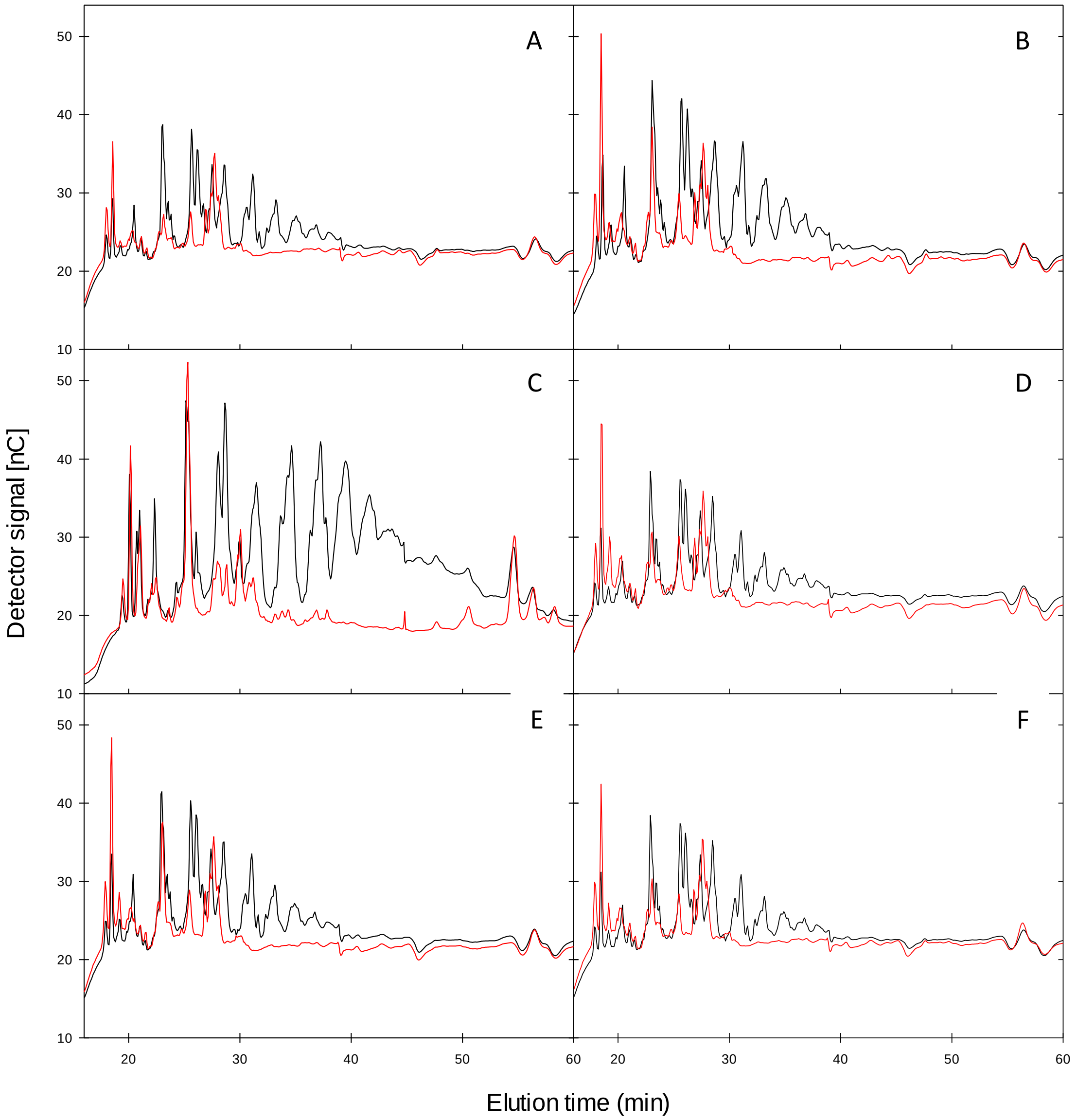

Supplementary Figure S1. HPAEC-PAD profiles of oligosaccharides after hydrolysis of starch from pea (**Panel A)**, fava bean ((**Panel B)**, potato (**Panel C)**, corn **(Panel D)**, wheat (**Panel E)** and barley (**Panel E)** by α- and β-amylases. Starches were treated with GlgB (black line); untreated starches served as control (red line). Monosaccharides eluting between 3 and 10 min are not shown.

Supplementary Table S1. Concentration of iodine-binding amylose in raw starches before and after the treatment with GlgB. Data are shown as average ± standard deviation of three replicate experiments. Values for GlgB treated starches are marked with an asterisk if treatment significantly ($P<0.05$) reduced the amylose content.

	**Amylose content (g/l)**	
	**Control**	**GlgB Treatment**
**Raw corn**	0.31 ± 0.07	0.24 ± 0.01
**Raw pea**	0.19 ± 0.02	0.14 ± 0.02*
**Raw wheat**	0.24 ± 0.04	0.22 ± 0.01
